# 

**Published:** 1906-06-09

**Authors:** 


					TEE HOSPITAL.
No. 1,028. Xol XL. [aCpfper] Satukday,
June 9th, 1906.
[-SPa.1*-] PRICE THREEPENCE

				

